# Neurons expressing the aryl hydrocarbon receptor in the locus coeruleus and island of Calleja major are novel targets of dioxin in the mouse brain

**DOI:** 10.1007/s00418-021-01990-1

**Published:** 2021-05-08

**Authors:** Eiki Kimura, Masanobu Kohda, Fumihiko Maekawa, Yoshiaki Fujii-Kuriyama, Chiharu Tohyama

**Affiliations:** 1 Laboratory of Environmental Health Sciences, Center for Disease Biology and Integrative Medicine, Graduate School of Medicine, The University of Tokyo, 7-3-1 Hongo, Bunkyo-ku, Tokyo, 113-0033 Japan; 2 Center for Health and Environmental Risk Research, National Institute for Environmental Studies, 16-2 Onogawa, Tsukuba, 305-8506 Japan; 3 Research Fellow, Japan Society for the Promotion of Science (JSPS), 5-3-1 Kojimachi, Chiyoda-ku, Tokyo, 102-0083 Japan; 4 Medical Research Institute, Molecular Epidemiology, Tokyo Medical and Dental University, 1-5-45 Yushima, Bunkyo-ku, Tokyo, 113-8510 Japan; 5 Faculty of Medicine, University of Tsukuba, 1-1-1 Tennodai, Tsukuba, 305-8575 Japan

**Keywords:** Aryl hydrocarbon receptor, Dioxin, Immunohistochemistry, Island of Calleja major, Locus coeruleus, Mouse

## Abstract

The aryl hydrocarbon receptor (AhR) acts as a receptor that responds to ligands, including dioxin. The AhR–ligand complex translocates from the cytoplasm into the nucleus to induce gene expression. Because dioxin exposure impairs cognitive and neurobehavioral functions, AhR-expressing neurons need to be identified for elucidation of the dioxin neurotoxicity mechanism. Immunohistochemistry was performed to detect AhR-expressing neurons in the mouse brain and confirm the specificity of the anti-AhR antibody using *Ahr*^−/−^ mice. Intracellular distribution of AhR and expression level of AhR-target genes, *Cyp1a1*, *Cyp1b1*, and *Ahr repressor* (*Ahrr*), were analyzed by immunohistochemistry and quantitative RT-PCR, respectively, using mice exposed to 2,3,7,8-tetrachlorodibenzo-*p*-dioxin (TCDD). The mouse brains were shown to harbor AhR in neurons of the locus coeruleus (LC) and island of Calleja major (ICjM) during developmental period in *Ahr*^+/+^ mice but not in *Ahr*^−/−^ mice. A significant increase in nuclear AhR of ICjM neurons but not LC neurons was found in 14-day-old mice compared to 5- and 7-day-old mice. AhR was significantly translocated into the nucleus in LC and ICjM neurons of TCDD-exposed adult mice. Additionally, the expression levels of *Cyp1a1*, *Cyp1b1*, and *Ahrr* genes in the brain, a surrogate of TCDD in the tissue, were significantly increased by dioxin exposure, suggesting that dioxin-activated AhR induces gene expression in LC and ICjM neurons. This histochemical study shows the ligand-induced nuclear translocation of AhR at the single-neuron level in vivo. Thus, the neurotoxicological significance of the dioxin-activated AhR in the LC and ICjM warrants further studies.

**Supplementary Information:**

The online version contains supplementary material available at 10.1007/s00418-021-01990-1.

## Introduction

The aryl hydrocarbon receptor (AhR), a ligand-activated transcription factor, exists in a wide range of animal species, including humans and rodents (Hahn 2002). Ligand-bound AhR translocates from the cytoplasm into the nucleus and enhances the expression of AhR-target genes, such as *Cyp1a1* and *Cyp1b1*, whereas these are not activated in *Ahr*^−/−^ mice (Mimura and Fujii-Kuriyama 2003). To date, evidence has accumulated to indicate the presence of endogenous and exogenous substances that act as AhR ligands (Barroso et al. 2021). An intake of indole-3-carbinol, a dietary AhR ligand, induces expression of *Cyp1a1* mRNA in the small intestine of the mouse (Li et al. 2011). Thus, intracellular localization of AhR can be directly linked to its transcriptional function.

Orthologues of the mammalian *Ahr* gene regulate neuronal growth in *Caenorhabditis elegans* and *Drosophila* (Huang et al. 2004; Kim et al. 2006; Qin and Powell-Coffman 2004; Smith et al. 2013). In rodents, *Ahr* transcripts are detected in various brain regions, including the cerebral cortex, cerebellum, hippocampus, and olfactory bulb (Kimura and Tohyama 2017; Petersen et al. 2000). In particular, *Ahr*^−/−^ mice show learning and memory impairments possibly due to atypical proliferation and morphology of hippocampal neurons (de la Parra et al. 2018; Latchney et al. 2013), implying that ligand-activated AhR is involved in the regulation of neuronal growth and brain function.

Exposure to dioxin, an exogenous AhR ligand, causes disease conditions, such as cleft palate and hydronephrosis, in *Ahr*^+/+^ mice but not *Ahr*^−/−^ mice (Mimura et al. 1997), showing that AhR is required for induction of dioxin toxicity. Disruption of neuronal migration and neurite elongation is observed in neurons expressing AhR constitutively in the mouse brain (Kimura et al. 2017), suggesting that AhR overactivation impairs neuronal growth and neural circuit structure. Indeed, perinatal dioxin exposure enhances AhR-target gene expression and alters neuromorphology in the mouse brain (Kimura et al. 2016, 2015). Furthermore, dioxin exposure adversely affects a variety of cognitive and neurobehavioral functions in humans (Nishijo et al. 2014; Patandin et al. 1999; Rogan et al. 1988) and rodents (Endo et al. 2012; Haijima et al. 2010; Kakeyama et al. 2014; Kimura et al. 2020; Kimura and Tohyama 2018). These results enabled us to speculate that brain neurons having a greater amount of AhR are strongly associated with dioxin neurotoxicity.

To understand the mechanism of dioxin neurotoxicity, we utilized histological experiments for the identification of AhR-rich neurons and analysis in intracellular AhR dynamics at the single-neuron level. We examined the expression of *Ahr* transcript and AhR protein in the mouse brain, identified AhR-expressing neurons immunohistochemically, and evaluated the nuclear translocation of AhR in dioxin-exposed mice.

## Materials and methods

### Animals

The experimental protocols were approved by the Animal Care and Use Committee of the University of Tokyo and that of the National Institute for Environmental Studies. Pregnant female and adult male C57BL/6J mice were purchased from CLEA Japan (Tokyo, Japan). *Ahr*^−/−^ mice with a B6.129S-Ahr < tm1Yfk > mouse strain (BRC01710) (Mimura et al. 1997) were provided by RIKEN BioResource Research Center (Tsukuba, Japan). *Ahr*^+/−^ male and female B6.129S-Ahr < tm1Yfk > mice were bred to obtain *Ahr*^−/−^ progeny. These mice were housed singly and in groups (three per cage), respectively, in an animal facility at a temperature of 22–24 °C and humidity of 40–60% on a 12:12-h light/dark cycle (lights on from 08:00 to 20:00). Laboratory rodent chow (Lab MR Stock; Nosan, Yokohama, Japan) and distilled water were provided ad libitum. Offspring were selected for transcript and protein expression analyses as described in sections of RT-PCR, western blotting, quantitative RT-PCR, and immunohistochemistry below. The number of animals used for these analyses is described in the legends to figures.

To produce and maintain the *Ahr*^*−/−*^ mouse strain, genotyping of the *Ahr* gene was performed as follows: genomic DNA was extracted from tail tips by lysis in 50 mM Tris–HCl (pH 8.0), 100 mM NaCl, 20 mM ethylenediaminetetraacetic acid (EDTA), 1% sodium dodecyl sulphate, and proteinase K (Wako Pure Chemicals, Osaka, Japan) at 55 °C for 4 h. The lysate was centrifuged at 17,400×*g* at 4 °C for 3 min. The genomic DNA in the supernatant was purified using phenol and chloroform, followed by washing with 70% ethanol. The genomic DNA (dissolved in Tris–EDTA buffer) was used as the template for PCR using the Takara LA Taq PCR kit (Takara Bio, Kusatsu, Japan) on a Veriti thermal cycler (Applied Biosystems, Foster City, CA, USA). The amplification conditions were as follows: 94 °C for 5 min, followed by 35 cycles of 94 °C for 30 s, 55 °C for 30 s, and 72 °C for 35 s. The PCR primers to amplify the genomic *Ahr* locus were 5′-GCCCGAGTCTCCTCTGTCG-3′/5′-CTCACGGCAGCGGAGATCT-3′ for the wild-type *Ahr* allele and 5′-GCCCGAGTCTCCTCTGTCG-3′/5′-CGCCGAGTTAACGCCATCAA-3′ for the *Ahr*-null allele. The 25-μl reaction contained 400 nM of each primer, 1× GC buffer II, 320 μM deoxynucleoside triphosphate (dNTP) mixture, and 0.5 U of LA Taq DNA polymerase. PCR products were separated by electrophoresis on agarose gels, which were stained with Midori Green Advance (Nippon Gene, Tokyo, Japan). The PCR products of the wild-type *Ahr* allele and *Ahr*-null allele were expected to be 439 and 671 bp in size, respectively.

### RT-PCR

Developing mice at P3 and P5 were decapitated, and several organs, including the brain, liver, lung, kidney, thymus, and spleen, were quickly removed and stored at −80 °C until RT-PCR analysis. The total RNA was isolated from each organ using an RNeasy Mini Kit (Qiagen, Tokyo, Japan). The cDNA for a given mRNA was synthesized using oligo-dT and random hexamers with a Primescript RT reagent kit (Takara Bio). Expression levels of *Ahr* and *Gapdh* transcripts were determined using a Veriti thermal cycler (Applied Biosystems) with a KOD Plus kit (Toyobo, Osaka, Japan). The amplification conditions were as follows: 95 °C for 1 min, followed by 35 cycles of 95 °C for 15 s, 55 °C for 15 s, and 68 °C for 30 s. The PCR primers for amplifying the murine *Ahr* and *Gapdh* transcripts were 5′-AGGATTTGCAAGAAGGAGAG-3′/5´-TTGGTTCGAATTTCCAGGAT-3´ and 5′-ACCCAGAAGACTGTGGATGG-3′/5′-CACATTGGGGGTAGGAACAC-3′, respectively. The 20-μl reaction solution contained 400 nM of each primer, 1× KOD Plus buffer, 200 μM dNTP mixture, 1 mM MgSO_4_, and 0.5 U of KOD Plus DNA polymerase. PCR products were separated by electrophoresis on agarose gels, which were stained with Midori Green Advance (Nippon Gene). The PCR products of the *Ahr* and *Gapdh* transcripts were expected to be 508 and 171 bp in size, respectively.

### Western blotting

Developing mice at P3, P5, and P14 were decapitated, and several organs (brain, liver, lung, kidney, thymus, and spleen) were quickly collected and stored at −80 °C until western blotting analysis. Protein was extracted at 4 °C in an ice bath unless stated otherwise. Each type of organ was homogenized with 4 mM HEPES–NaOH buffer, pH 7.3, containing 0.32 M sucrose and 1% protease inhibitor cocktail (Sigma-Aldrich, St. Louis, MO, USA), using a Potter-type homogenizer. The homogenates were centrifuged at 1000×*g* at 4 °C for 10 min, and the supernatants were used for western blotting. Protein concentration in the supernatants was measured with the Quick Start Bradford Protein Assay (BioRad, Hercules, CA, USA). Proteins in the supernatants were separated on a 7.5% polyacrylamide gel and blotted onto immobilon-P transfer membranes (Millipore, Bedford, MA, USA). The proteins adsorbed to membranes were allowed to react with mouse monoclonal anti-AhR antibody (1:1000; sc-398877, Santa Cruz Biotechnology, Santa Cruz, CA, USA) in Tris-buffered saline, pH 7.4, containing 0.1% Tween-20 (TBST), overnight at 4 °C, followed by incubation in TBST containing anti-mouse IgG-horseradish peroxidase (HRP)-conjugated antibody (1:5000; 7076S, Cell Signaling Technology, Beverly, MA, USA), for 1 h at room temperature. Chemi-Lumi One (Nacalai Tesque, Kyoto, Japan) was used to visualize the protein bands, which were detected on Hyperfilm ECL (GE Healthcare Ltd., Tokyo, Japan) and developed and fixed with GBX developer and GBX fixer (Kodak, Rochester, NY, USA), respectively. Following deactivation of endogenous HRP by incubation in TBST containing 15% hydrogen peroxide for 30 min at room temperature, the membranes were immersed in TBST containing rabbit polyclonal anti-GAPDH antibody (1:5000; ab9485, Abcam, Cambridge, UK), overnight at 4 °C, followed by incubation in TBST containing anti-rabbit IgG-HRP-conjugated antibody (1:5000; 7074S, Cell Signaling Technology). Then, targeted protein bands were visualized in the same manner as described for AhR detection. The intensity of AhR and GAPDH bands was measured using ImageJ software (National Institutes of Health, Bethesda, MD, USA).

### Chemical treatment

2,3,7,8-tetrachlorodibenzo-*p*-dioxin (TCDD; purity > 99.5%) was purchased from Cambridge Isotope Laboratory (Andover, MA, USA). Corn oil and *n*-nonane were purchased from Wako Pure Chemicals and Nacalai Tesque, respectively. Twelve-week-old male C57BL/6J mice were divided to control and TCDD groups, and they were orally administered with vehicle (corn oil containing 0.6% *n*-nonane) or TCDD dissolved in vehicle (20 μg/kg body weight).

### Quantitative RT-PCR

Brain and liver tissues of 12-week-old male mice treated with vehicle or TCDD were collected quickly and stored at −80 °C until analysis. Total RNA was isolated from the brain and liver using an RNeasy Mini Kit (Qiagen). The cDNA for a given mRNA was synthesized using oligo-dT and random hexamer primers with a PrimeScript RT reagent kit (Takara). Gene expression levels were determined quantitatively using a LightCycler System (Roche Molecular Biochemicals, Indianapolis, IN, USA) with Thunderbird SYBR qPCR Mix (Toyobo). The genes and primers are summarized in Supplementary Table 1. No-template reactions were analyzed in every PCR to monitor for cross-contamination. To verify the specificity of amplification, melting curve analyses of the products were performed at the end of every PCR. The *Cyp1a1*, *Cyp1b1*, and *Ahr repressor* (*Ahrr*) mRNA expression levels were calculated using the ΔΔC_t_ method and normalized to the *18S rRNA* expression.

### Immunohistochemistry

Developing and adult mice were transcardially perfused with 4% paraformaldehyde in 0.1 M phosphate-buffered saline (PBS, pH 7.4) under anesthesia with sodium pentobarbital (conducted at the University of Tokyo) or three types of mixed anesthetic agents containing medetomidine hydrochloride, midazolam, and butorphanol (at the National Institute for Environmental Studies). Brains were collected, fixed in 4% paraformaldehyde overnight, immersed in a series of 0.1 M PBS containing 5%, 15%, and 30% sucrose, frozen in Tissue-Tek O.C.T. compound (Sakura Finetek, Tokyo, Japan), and stored at −80 °C until histological sectioning. Frozen brains were sliced in the sagittal plane using a cryostat (Model 3050S; Leica Microsystems, Tokyo, Japan). Brain sections were cut at 50 μm thickness for immunofluorescence analysis.

Brain tissue sections were immunohistochemically stained for AhR, tyrosine hydroxylase (TH), dopamine β-hydroxylase (DBH), or NeuN. In brief, the tissue sections were washed in PBS containing 0.1% Triton X-100 (PBST), soaked in 0.01 M citrate buffer (pH 6.0) (Muto Pure Chemicals, Tokyo, Japan), and incubated at 90 °C (developing mouse brains) or 65 °C (adult mouse brains) in a water bath for 10 min. The sections were blocked with PBST containing 5% bovine serum albumin (A3059; Sigma-Aldrich) (blocking solution) and allowed to react with mouse monoclonal anti-AhR antibody (1:500; sc-398877, Santa Cruz Biotechnology) and rabbit polyclonal anti-TH antibody (1:1000; ab112, Abcam), anti-DBH antibody (1:1000; 22,806, Immunostar, Hudson, WI, USA), or anti-NeuN (1:1000; ab177487, Abcam) in blocking solution overnight at 4 °C. Then, the signals of AhR and TH, DBH, or NeuN were visualized with the respective secondary antibodies anti-mouse IgG AlexaFluor 488 (Life Technologies, Gaithersburg, MD, USA) and anti-rabbit IgG AlexaFluor 568 (Life Technologies) in PBST (1:1000). Furthermore, the nucleus was stained with PBST containing Hoechst 33342 (1:1000; Dojin Laboratories, Kumamoto, Japan), followed by mounting with VECTASHIELD (H-1400; Vector Laboratories, Burlingame, CA, USA) for confocal microscopy. Immunostained images were captured using an inverted Leica DMi8 microscope, equipped with the Leica TCS SP8 confocal module (Leica Microsystems). Specific objective lens (HC PL APO CS 10×/NA = 0.40 and HC PL APO CS2 20×/NA = 0.75; Leica Microsystems) and LAS X 3.1.5 software (Leica Microsystems) were used to capture images (*x* = 2048 pixels and *y *= 2048 pixels, bit depth = 8 in each RGB color).

### Intracellular localization of AhR

Cellular morphology and immunostaining intensity of AhR- and TH-double-positive cells in the locus coeruleus (LC) and AhR- and NeuN-double-positive cells in the island of Calleja major (ICjM) were analyzed by applying the ImageJ software to the confocal microscopy images. In analyses of AhR- and TH-double-positive cells in the LC, we outlined the nucleus and soma of TH-positive cells [i.e., noradrenergic (NA) neurons] and measured their nuclear and soma sectional areas, and then calculated the nuclear area percentage by dividing the nuclear area by the soma area in each cell. In addition, we determined AhR immunostaining intensity per nucleus and soma (AhR^Nuc^ intensity and AhR^Soma^ intensity, respectively) of TH-positive cells. After the background subtraction of AhR-stained images, the AhR^Nuc^ intensity percentage was calculated by dividing the AhR^Nuc^ intensity by the AhR^Soma^ intensity. In order to analyze the intracellular localization of AhR, AhR^Nuc^ intensity percentage data were normalized depending on the soma size of TH-positive cells. We divided the AhR^Nuc^ intensity percentage by the nuclear area percentage to calculate the ratio in locus coeruleus-noradrenergic (LC-NA) neurons (ratio^LC−NA^, thereafter). In order to compare ratio^LC−NA^ of individual mice between the control and TCDD groups, we used the distribution of ratio^LC−NA^ values in each mouse as a surrogate parameter and analyzed the percentage of ratio^LC−NA^ that was divided by the arbitrarily chosen value of 0.2. For each mouse, 57 to 103 cells in developing mice and 51 to 96 cells in adult mice were subjected to analyses in intracellular localization of AhR in TH-positive cells in the LC. In analyses of AhR- and NeuN- double-positive cells in the ICjM, we outlined the nucleus of NeuN-positive cells and measured nuclear sectional areas and AhR^Nuc^ intensity in each cell. To adjust the variability of luminance among images, AhR^Nuc^ intensity was normalized to the mean value of immunostained AhR intensity in the whole ICjM area. The parameter ratio in ICjM neurons (ratio^ICjM^, thereafter) was calculated by dividing AhR^Nuc^ intensity by the nuclear area in each cell. Furthermore, we analyzed the percentage of the ratio^ICjM^ that was divided by the arbitrarily chosen value of 0.005. The numbers of cells that were used to analyze the intracellular localization of AhR in NeuN-positive cells in the ICjM ranged from 65 to 263 cells in developing mice and from 133 to 291 cells in adult mice.

### Statistical analysis

Statistical analysis was performed using BellCurve for Excel software (Social Survey Research Information Co., Ltd., Tokyo, Japan). Protein expression, cellular morphology, immunostaining intensity, and ratio values were analyzed using Student’s *t*-test or one-way analysis of variance (ANOVA), followed by the Tukey–Kramer post hoc test, and *p*-values < 0.05 were considered statistically significant. Because the mention of *F*- and *p*-values for each statistical analysis in the main text is very complicated, statistically significant differences are shown by asterisks in each figure.

## Results

### AhR expression in developing organs

**Fig. 1:**
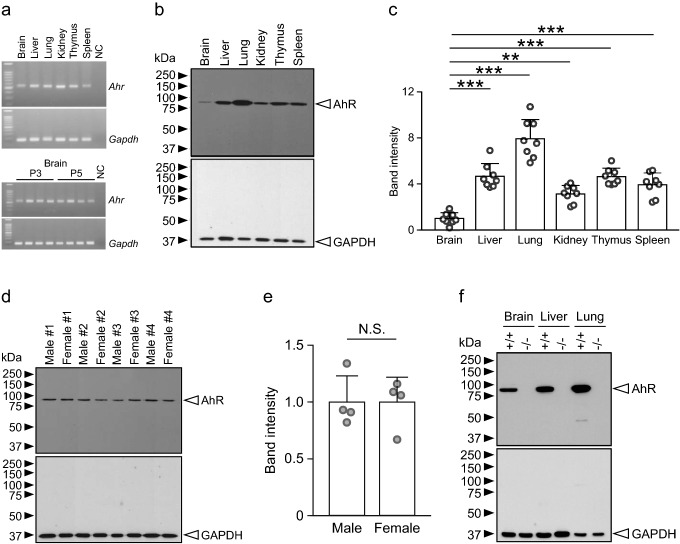
*Ahr* transcript and AhR protein expression in developing mouse organs. **a** RT-PCR–amplified *Ahr* (508 bp) and *Gapdh* (171 bp) transcripts were detected in the brain, liver, lung, kidney, thymus, and spleen of mice at P3 and P5 (*n* = 4 mice at each stage). *NC* negative control. **b** Representative images showing AhR and GAPDH proteins detected by western blotting in the brain, liver, lung, kidney, thymus, and spleen of male mice at P3. **c** Quantitative analysis of AhR band intensity in six organs (*n* = 8 mice/tissue). **d** Images showing AhR and GAPDH proteins detected by western blotting in the brains of male and female mice at P5. **e** Quantitative analysis of AhR band intensity in male and female mouse brains (*n* = 4 mice/group) at P5, showing no significant difference in AhR protein amounts between sexes. AhR band intensity was normalized to GAPDH band intensity. **f** Images showing AhR and GAPDH proteins detected by western blotting in the brain, liver, and lung of *Ahr*^+/+^ and *Ahr*^−/−^ mice at P5 (*n* = 1 in each genotype). No AhR protein was detected in these tissues of *Ahr*^−/−^ mice, demonstrating the specificity of the antibody. *Circles* represent individual mouse data. Values are shown as the mean ± SD. *Asterisks* (** and ***) denote statistical significance at *p* < 0.01 and 0.001, respectively, by one-way ANOVA with the Tukey–Kramer post hoc test

In developing mice, AhR was observed in various organs, including the brain, by RT-PCR and western blotting, although AhR protein amounts in the brain were significantly lower than those in other organs (Fig. 1a–c). No significant difference in expression was found between male and female brains (Fig. 1d, e). The AhR protein was not detected in *Ahr*^−/−^ mice (Fig. 1f), indicating the specificity of the anti-AhR antibody.

### AhR localization in neurons of the developing brain

**Fig. 2:**
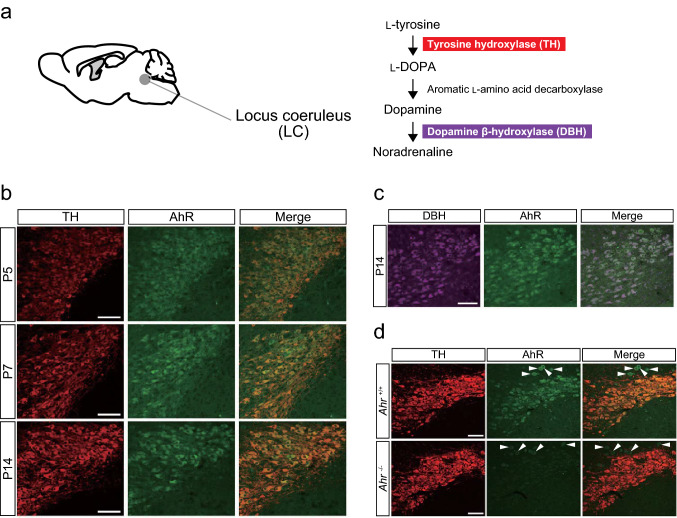
AhR expression in mouse LC-NA neurons at P5, P7, and P14. **a** Diagram displaying the location of the LC (left) and the metabolic pathway of noradrenaline synthesis in LC-NA neurons (right). **b**, **c** Brain tissue sections immunostained with anti-TH or DBH and anti-AhR antibodies. AhR was detected in TH-expressing neurons in the LC at P5, P7, and P14 (*n* = 3 mice at each stage) (**b**). Additionally, AhR was also found in neurons expressing DBH in the LC at P14 (*n* = 3 mice) (**c**). Scale bar = 100 μm. **d** Representative images showing the LC of *Ahr*^+/+^ and *Ahr*^−/−^ mice at P14 (*n* = 3 mice in each genotype), demonstrating antibody specificity. Arrowheads mark TH-negative cells with nonspecific anti-AhR antibody staining. Scale bar = 100 μm

**Fig. 3:**
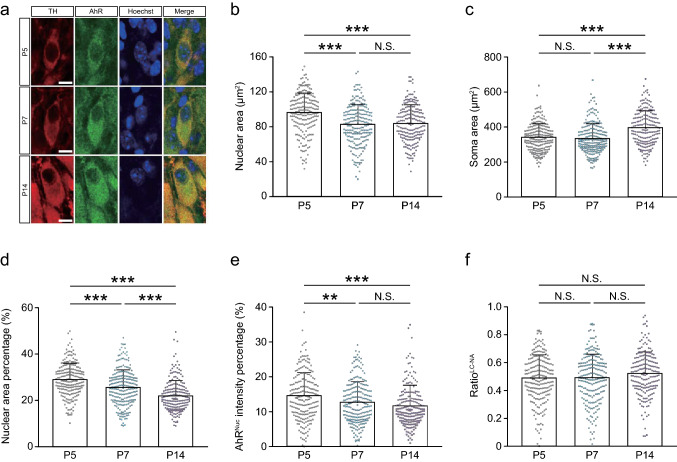
Intracellular localization of AhR in mouse LC-NA neurons at P5, P7, and P14. **a** Immunostained LC-NA neurons double-positive for TH and AhR. The cellular boundary of TH-stained cells was clearly observed, which enabled the measurement of the nuclear and soma areas of individual LC-NA neurons. Scale bar = 10 μm. **b**–**f** Quantitative analyses of AhR-expressing LC-NA neurons. The nuclear area was significantly larger at P5 than at P7 and P14 (**b**), whereas the soma area was significantly larger at P14 compared to P5 and P7 (**c**), indicating that marked changes in cellular morphology occur between P5 and P14. The nuclear area percentage that represents the nuclear area normalized to the soma area in each neuron was significantly decreased during the period from P5 to P14 (**d**). The AhR immunostaining intensity per nucleus (AhR^Nuc^ intensity) was normalized to that of the soma in each neuron (AhR^Nuc^ intensity percentage). The AhR^Nuc^ intensity percentage at P5 was significantly higher than that at P7 and P14 (**e**). To normalize the developmental-stage-related changes in cellular morphology, the ratio calculated by dividing AhR^Nuc^ intensity percentage by nuclear area percentage (ratio^LC−NA^) served as an index to evaluate the nuclear AhR. No significant difference in ratio^LC−NA^ was found across developmental periods (**f**). Values are shown as the mean ± SD. *Circles* represent individual cell data (215, 228, and 200 cells from 3 mice each at P5, P7, and P14, respectively). *Asterisks* (** and ***) denote statistical significance at *p* < 0.01 and 0.001, respectively, by one-way ANOVA with the Tukey–Kramer post hoc test

Distinct AhR immunostaining was observed in the LC, where AhR was detected in nearly all the NA neurons expressing TH or DBH in *Ahr*^+/+^ mice at P5, P7, and P14, whereas no AhR was detected in *Ahr*^−/−^ mice (Fig. 2a–d and Supplementary Table 2). Because the nuclear and soma areas of LC-NA neurons increased differently between P5 and P14 (Fig. 3a–c), two indexes were used for a normalization purpose: the percentage of nuclear area to soma area and the percentage of AhR^Nuc^ intensity to AhR^Soma^ intensity. The nuclear area percentage decreased significantly with age (P5, P7, and P14; Fig. 3d). Despite a significantly higher AhR^Nuc^ intensity at P5 than P7 and P14 (Fig. 3e), no significant difference was found in the normalized ratio of AhR^Nuc^ intensity percentage to nuclear area percentage (ratio^LC−NA^) during development (Fig. 3f).

**Fig. 4:**
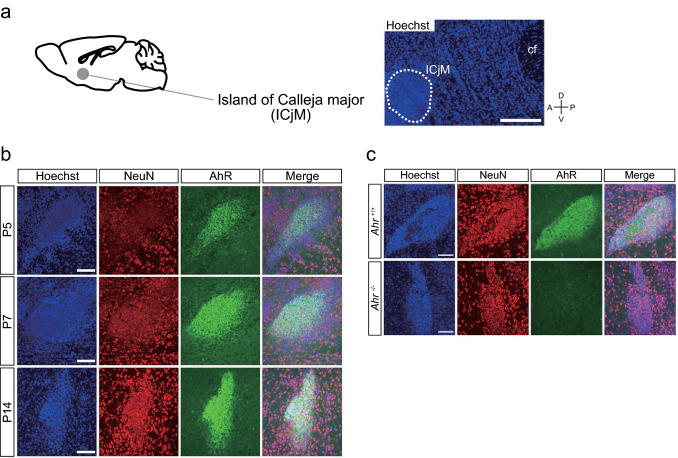
AhR expression in mouse ICjM neurons at P5, P7, and P14. **a** Diagram (left) illustrating the location of the ICjM anterior to the commissural fiber (cf). A representative low-magnification image (right) of Hoechst-stained brain tissue at P5 is shown. Scale bar = 200 μm. **b** Representative images of immunostained sections of the ICjM at P5, P7, and P14 show distinct AhR signals in cells expressing NeuN, a marker of mature neurons (*n* = 3 mice at each stage). Scale bar = 100 μm. **c** Representative images showing the ICjM of *Ahr*^+/+^ and *Ahr*^−/−^ mice at P14 (*n* = 3 mice in each genotype). Immunostained AhR signals were not observed in *Ahr*^−/−^ mice, demonstrating the specificity of the antibody. Scale bar = 100 μm

**Fig. 5:**
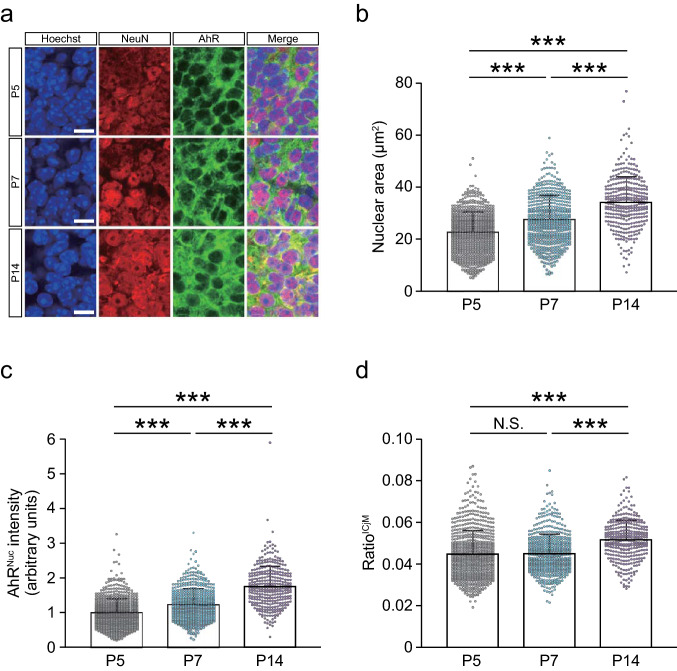
Intracellular localization of AhR in mouse ICjM neurons at P5, P7, and P14. **a** Immunostained ICjM neurons double-positive for NeuN and AhR. Because the cellular boundary could not be distinctly visualized, the soma areas of each neuron were not characterized. Scale bar = 10 μm. **b**–**d** Quantitative analyses of AhR-expressing ICjM neurons. The nuclear area of each neuron gradually increased with brain development (**b**). The AhR immunostaining intensity per nucleus (AhR^Nuc^ intensity) was significantly increased during the period from P5 to P14 (**c**). The ratio^ICjM^ representing AhR^Nuc^ intensity normalized to the nuclear area was significantly higher at P14 than those at P5 and P7, and no significant difference was observed between P5 and P7 (**d**). Values are shown as the mean ± SD. *Circles* represent individual cell data (711, 528, and 332 cells from 3 mice at each stage, P5, P7, and P14, respectively). *Asterisks* (***) denote statistical significance at *p* < 0.001 by one-way ANOVA with the Tukey–Kramer post hoc test

We also found AhR in cells expressing NeuN, a neuronal marker, in the ICjM of *Ahr*^+/+^ mice, but not in *Ahr*^−/−^ mice (Fig. 4a–c). The boundary of the soma area of ICjM neurons was not traceable because they are densely located (Fig. 5a). Thus, using the nuclear area, we normalized the AhR^Nuc^ intensity to get an index (ratio^ICjM^). The AhR^Nuc^ intensity was found to vary at the three different ages, and the ratio^ICjM^ was significantly higher at P14 than at P5 and P7 (Fig. 5b–d).

To study AhR dynamics in other brain regions, we analyzed the AhR expression in the cerebral cortex, cerebellum, hippocampus, and olfactory bulb at P14. Although AhR in these regions was observed by western blotting, distinct immunohistochemical signals were not detected (Supplementary Fig. 1a–c).

### Nuclear translocation of AhR in dioxin-exposed mice

**Fig. 6:**
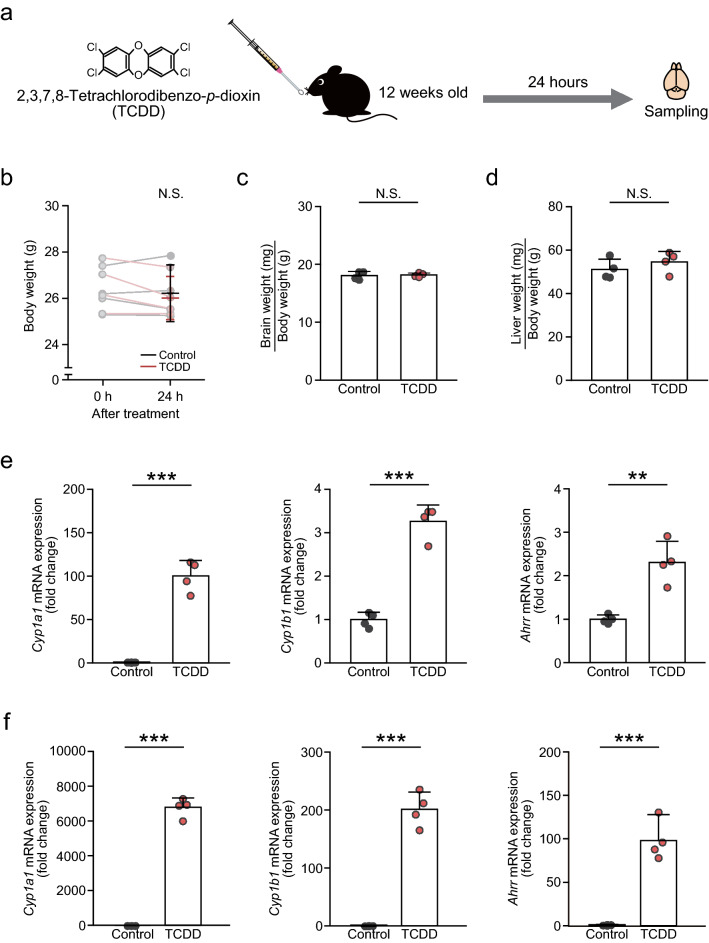
Expression level of AhR-target genes in the brain and liver of TCDD-exposed mice. **a** Schematic of the TCDD experiment. Twelve-week-old mice were orally exposed to TCDD (20 μg/kg body weight), and their brains and livers were sampled 24 h later. The liver was used as a positive control to confirm increased expression of the AhR-target genes *Cyp1a1*, *Cyp1b1*, and *Ahrr*, which are drastically enhanced by TCDD exposure. **b**–**d** Body weight **b** and organ size of the brain **c** and liver **d** in the control and TCDD groups. Upon TCDD exposure, no changes in these weights were observed between the two groups. **e**, **f** Expression levels of *Cyp1a1*, *Cyp1b1*, and *Ahrr* mRNAs in the brain **e** and liver **f**. In the TCDD group, the expression of the three AhR-target genes in the brain and liver was significantly increased. *Circles* represent individual mouse data (*n* = 4 mice/group). Values are shown as the mean ± SD. *Asterisks* (** and ***) denote statistical significance at *p* < 0.01 and 0.001, respectively, by Student’s *t*-test

We studied whether dioxin altered the intracellular localization of AhR in LC-NA and ICjM neurons. Adult mice treated with TCDD, the most toxic dioxin congener (Van den Berg et al. 2006), did not show any signs of weight loss (Fig. 6a–d). In the TCDD group, the expression levels of the AhR-target genes *Cyp1a1*, *Cyp1b1*, and *Ahrr* were significantly increased in the brain and liver (Fig. 6e, f), indicating nuclear translocation of AhR upon dioxin exposure.

**Fig. 7:**
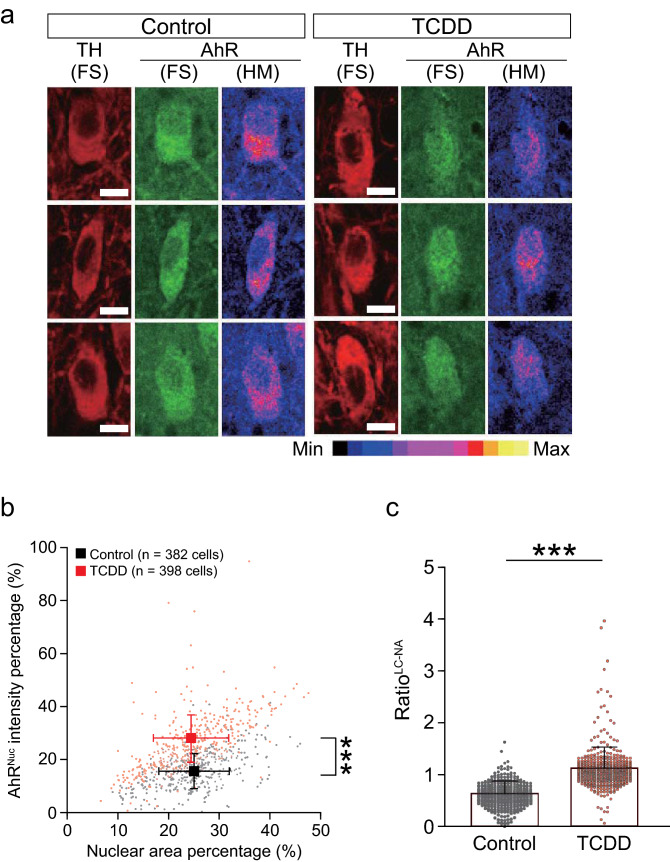
Nuclear translocation of AhR in LC-NA neurons of TCDD-exposed mice. **a** Representative images showing fluorescent signal (FS) of TH and AhR in LC-NA neurons in the control and TCDD groups. Heatmap (HM) images represent the relative intensity of immunostained AhR signals. The fluorescent intensity scale for HM images is shown below the images. Scale bar = 10 μm. **b** Scatter plot of nuclear area percentage (*x*-axis) and AhR^Nuc^ intensity percentages (*y*-axis) in LC-NA neurons in the control (*black*) and TCDD (*red*) groups. The AhR^Nuc^ intensity percentage of the TCDD group was significantly higher than that of the control group without a significant change in the nuclear area percentage. **c** The ratio^LC−NA^ was significantly higher in the TCDD group than in the control group. Values are shown as the mean ± SD. *Circles* represent individual cell data (382 and 398 cells from 6 mice each in the control and TCDD groups, respectively). *Asterisks* (***) denote statistical significance at *p* < 0.001 by Student’s *t*-test

**Fig. 8:**
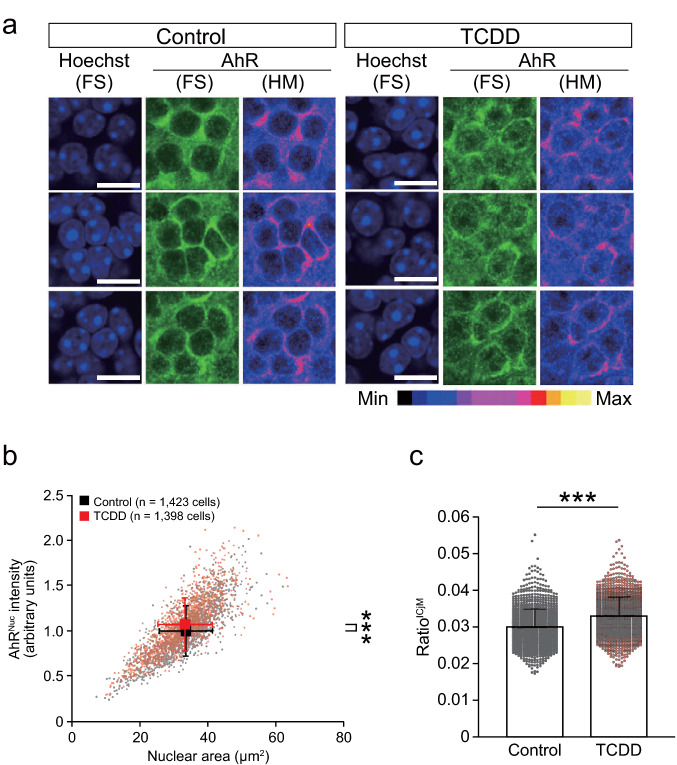
Nuclear translocation of AhR in ICjM neurons of TCDD-exposed mice. **a** Representative images showing fluorescent signal (FS) of Hoechst and AhR in ICjM neurons in the control and TCDD groups. Heatmap (HM) images represent the relative intensity of immunostained AhR signals. The fluorescent intensity scale for HM images is shown below the images. Scale bar = 10 μm. **b** Scatter plot of nuclear area (*x*-axis) and AhR^Nuc^ intensity (*y*-axis) in ICjM neurons in the control (*black*) and TCDD (*red*) groups. The AhR^Nuc^ intensity of the TCDD group was significantly higher than that of the control group without a significant change in the nuclear area. **c** The ratio^ICjM^ was significantly higher in the TCDD group than in the control group. Values are shown as the mean ± SD. *Circles* represent individual cell data (1423 and 1398 cells from six mice each in the control and TCDD groups, respectively). *Asterisks* (***) denote statistical significance at *p* < 0.001 by Student’s *t*-test

We observed no significant difference in the nuclear area of LC-NA neurons between the control and TCDD groups (Fig. 7a and Supplementary Fig. 2a, b). Although the soma area in the TCDD group was significantly higher than that in the control group (Supplementary Fig. 2c), the normalized nuclear area percentage did not differ (Fig. 7b), suggesting the minimized toxicity, if any, at the cellular level. The AhR^Nuc^ intensity percentage and ratio^LC−NA^ were significantly increased in the TCDD group (Fig. 7b, c). To examine the effects of TCDD exposure on individual mice, we also compared the two groups regarding the distribution of ratio^LC−NA^ values in each mouse and found that they were significantly shifted toward higher values in the TCDD group compared to control (Supplementary Fig. 2d). In ICjM neurons, both AhR^Nuc^ intensity and ratio^ICjM^ in the TCDD group were significantly higher than those in the control group without a significant change in the nuclear area (Fig. 8a–c and Supplementary Fig. 3a). The distribution of ratio^ICjM^ values in individual mice was right-shifted toward higher values in the TCDD group compared to control (Supplementary Fig. 3b). These histological analyses showed the translocation of AhR from the cytoplasm into the nucleus in the LC-NA and ICjM neurons of TCDD-exposed mice.

## Discussion

The AhR–ligand complex translocates into the cellular nucleus to enhance the expression of AhR-target genes, which in turn may induce developmental and physiological responses or toxicities. Thus, the types of brain neurons expressing AhR must be characterized for understanding the impacts of AhR ligands on the nervous system. In the present study, we immunohistochemically identified two neuronal populations having AhR in the mouse LC and ICjM and quantitatively analyzed nuclear translocation of AhR at the single-neuron level. Although AhR expression in neurons and glias has been reported in humans and rodents (Bravo-Ferrer et al. 2019; de la Parra et al. 2018; Rothhammer et al. 2018, 2016; Shackleford et al. 2018), the specificity of the AhR antibodies in these studies has not been verified by immunohistochemistry using AhR-null tissues. After confirming the specificity of the AhR antibody using *Ahr*^*−/−*^ mouse brains, we unequivocally demonstrated the presence of AhR in LC-NA and ICjM neurons (Figs. 2d, 4c). Furthermore, a significant increase in nuclear AhR was found in these neurons of TCDD-exposed mice (Figs. 7, 8 and Supplementary Figs. 2, 3), which is consistent with gene expression changes (Fig. 6e). Thus, our immunohistochemical analysis is considered to be robust. We describe three implications of the presence of neuronal AhR in the LC and ICjM below.

First, loss-of-function and gain-of-function experiments reveal that AhR regulates neurogenesis, neuronal migration, and neurite elongation in *C. elegans* (Huang et al. 2004; Qin and Powell-Coffman 2004; Smith et al. 2013), *Drosophila* (Kim et al. 2006), and mice (de la Parra et al. 2018; Kimura et al. 2017; Latchney et al. 2013). Thus, it is plausible that AhR is involved in the growth of LC-NA and ICjM neurons. Although it has been reported that neurogenesis of ICjM occurs in rat fetuses (Bayer 1985) and that ICjM structure is formed in mice at P2 (Hsieh and Puche 2013), the molecular mechanisms regulating the growth of ICjM neurons remain largely unclear. Since, in the present study, altered AhR dynamics was found in the nuclei of ICjM neurons during development (Fig. 5), further studies on the role of AhR in ICjM neurons could help understand the mechanism of the ICjM formation. On the other hand, no distinct AhR immunostaining was observed in the cerebral cortex, cerebellum, hippocampus, and olfactory bulb, where AhR protein was detected by western blotting (Supplementary Fig. 1). One plausible explanation would be that AhR abundance in LC-NA and ICjM neurons is greater than that in other neurons, suggesting the possibility of AhR as a marker for specified neuronal populations.

Second, the intracellular dynamics of AhR is essential for understanding how AhR ligands impact cellular activities. AhR ligands contained in diet and gut microbiota metabolites have been reported to regulate various physiological systems. Treatment with indole-3-carbinol enhances the immune capacity of *Ahr*^+/+^ mice but not *Ahr*^−/−^ mice (Kiss et al. 2011; Li et al. 2011). Furthermore, indole-3-aldehyde produced by lactobacilli has AhR agonistic activity and protects against candidiasis and colitis in an AhR-dependent manner (Zelante et al. 2013). In particular, a large body of evidence suggests that gut microbiota influences neuronal activities and brain functions (Mayer et al. 2015). However, the molecular mechanisms linking gut microbiota with brain neurons are not fully understood, although several pathways via which microbiota affect brain function have been proposed (Cryan and Dinan 2012). Our present study provides experimental evidence that oral exposure to TCDD significantly increases nuclear translocation of AhR in LC-NA and ICjM neurons (Figs. 7, 8 and Supplementary Figs. 2, 3), suggesting that other AhR ligands might also influence the AhR dynamics and signaling activation in these neurons.

Third, cognitive impairments and neurobehavioral abnormalities have been reported in humans and laboratory animals perinatally exposed to dioxin (Endo et al. 2012; Haijima et al. 2010; Kakeyama et al. 2014; Kimura et al. 2020; Kimura and Tohyama 2018; Nishijo et al. 2014; Patandin et al. 1999; Rogan et al. 1988). However, the brain regions and neuronal populations responsible for those neurotoxic effects remain still uncharacterized. Our immunohistochemical analysis demonstrated that LC-NA and ICjM neurons are targets of dioxin (Figs. 7, 8 and Supplementary Figs. 2, 3). LC-NA neurons elongate their axons into a wide range of brain regions and regulate a variety of brain functions (Waterhouse and Navarra 2019). For example, in rodents, the LC is involved in sleep/awake states (Aston-Jones and Bloom 1981), stress response (Ziegler et al. 1999), behavioral flexibility (McGaughy et al. 2008), fear memory (Soya et al. 2013), and everyday memory (Takeuchi et al. 2016) as well as infant attachment learning (Moriceau et al. 2009). Remarkably, mouse offspring born to dams exposed to TCDD show abnormalities related to attachment behavior in infancy (Kimura and Tohyama 2018) and executive function and emotion in adulthood (Endo et al. 2012; Haijima et al. 2010), suggesting that these phenotypes could be caused by impaired growth of LC-NA neurons. Additionally, a change in the number of midbrain dopaminergic neurons in TCDD-exposed mice (Tanida et al. 2009) has been reported. ICjM neurons receive axonal projections from midbrain dopaminergic neurons (Fallon et al. 1978) and express dopamine receptors (Mengod et al. 1991; Sokoloff et al. 1990). The dopaminergic circuit plays a role in reward-related behavior (Ikemoto 2007), and rats afflicted with drug addiction show an increase in ICjM neuronal activity (Prast et al. 2014; Singh et al. 2006), suggesting an involvement of the ICjM in reward-related behavior. Thus, it is plausible that dioxin adversely affects the growth of both ICjM and dopaminergic neurons, leading to an abnormality in reward-related behavior. Collectively, our histological results highlight the need for studies focusing on LC-NA and ICjM neurons to understand the molecular mechanisms of dioxin neurotoxicity.

## Supplementary Information

Below is the link to the electronic supplementary material.

Supplementary file1 (PDF 494 KB)

## Data Availability

The data that support the findings of this study are available from the corresponding author upon reasonable request.
