# Supplementary material for: Neurons expressing the aryl hydrocarbon receptor in the locus coeruleus and island of Calleja major are novel targets of dioxin in the mouse brain
Source: Histochem Cell Biol. 2021 May 8;156(2):147–63. doi: 10.1007/s00418-021-01990-1 (PMC8397641; doi:10.1007/s00418-021-01990-1)
Supplement: Supplementary file 1 — Supplementary file1 (PDF 494 KB) [file 418_2021_1990_MOESM1_ESM.pdf]

Supplementary Information

Neurons expressing the aryl hydrocarbon receptor in the locus coeruleus and island of Calleja major are novel targets of dioxin in the mouse brain

Eiki Kimura[1, 2, 3], Masanobu Kohda[1, 2], Fumihiko Maekawa[2], Yoshiaki Fujii-Kuriyama[4], Chiharu Tohyama[1, 2, 5]

[1] Laboratory of Environmental Health Sciences, Center for Disease Biology and Integrative Medicine, Graduate School of Medicine, The University of Tokyo, 7-3-1 Hongo, Bunkyo-ku, Tokyo 113-0033, Japan

[2] Center for Health and Environmental Risk Research, National Institute for Environmental Studies, 16-2 Onogawa, Tsukuba, 305-8506, Japan

[3] Research Fellow, Japan Society for the Promotion of Science, 5-3-1 Kojimachi, Chiyoda-ku, Tokyo 102-0083, Japan

[4] Medical Research Institute, Molecular Epidemiology, Tokyo Medical and Dental University, 1-5-45 Yushima, Bunkyo-ku, Tokyo 113-8510, Japan

[5] Faculty of Medicine, University of Tsukuba, 1-1-1 Tennodai, Tsukuba, 305-8575, Japan

Corresponding authors:

Eiki Kimura, Center for Health and Environmental Risk Research, National Institute for Environmental Studies, 16-2 Onogawa, Tsukuba, 305-8506, Japan.

Telephone: +81-29-850-2402.

Email: eiki-kimura@umin.ac.jp

Chiharu Tohyama, Faculty of Medicine, University of Tsukuba, 1-1-1 Tennodai, Tsukuba, 305-8575, Japan.

Telephone: +81-29-853-3016

Email: tohyama.chiharu@hestic.com

Supplementary Table 1. Primer sequences for quantitative RT-PCR.

Gene symbol	Forward primer	Reverse primer
18S rRNA	5′-GGACCAGAGCGAAAGCATTTG-3′	5′-TTGCCAGTCGGCATCGTTTAT-3′
Ahrr	5′-CAGGGCAGACATTGTGGTTA-3′	5′-CTCCATTGCTCTTTCCTGCT-3′
Cyp1a1	5′-CACCGTATTCTGCCTTGGAT-3′	5′-CAGCATGTGACCAATGAAGG-3′
Cyp1b1	5′-GGACAAGGACGGCTTCATTA-3′	5′-GCGAGGATGGAGATGAAGAG-3′

Supplementary Table 2. Percentage of TH- and AhR-double-positive cells relative to TH-positive cells in the LC.

Age (number of mice)	Total number of cells		Percentage of TH- and AhR-double-positive cells to TH-positive cells†
	TH-positive	TH- and AhR-double-positive	
P5 (n = 6)	594	576	96.5 ± 5.1%
P7 (n = 6)	623	619	99.2 ± 1.0%
P14 (n = 5)	517	513	99.1 ± 1.6%

† Values are shown as the mean ± SD.

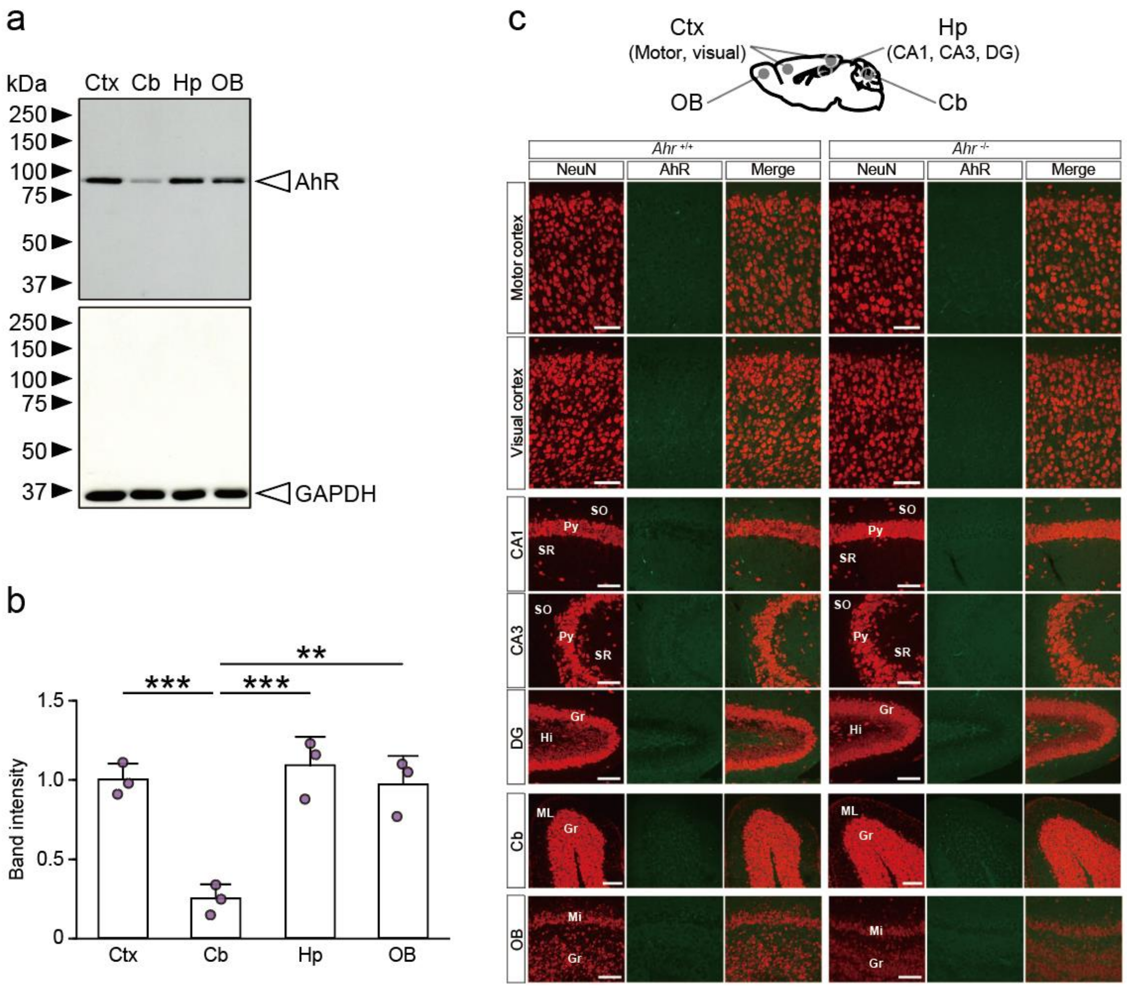

Supplementary Figure 1. AhR protein expression analysis in the cerebral cortex, cerebellum, hippocampus, and olfactory bulb. (a) Representative images of AhR and GAPDH proteins detected by western blotting in the cerebral cortex (Ctx), cerebellum (Cb), hippocampus (Hp), and olfactory bulb (OB) at P14. (b) Quantitative analysis of AhR band intensity in these four brain regions. AhR amounts in the Ctx, Hp, and OB were greater than that in the Cb. AhR band intensity was normalized to GAPDH band intensity. Circles represent individual data (n = 3 mice/region). (c) Representative images of NeuN- and AhR-stained Ctx, Cb, Hp, and OB regions of *Ahr*$^{+/+}$ and *Ahr*$^{-/-}$ mice at P14 (n = 3 mice in each genotype). The diagram identifies the regions sampled. No distinct immunostained AhR signal was observed in these brain regions. Scale bars = 100 μm. Gr, granule cell layer; Hi, hilus; Mi, mitral cell layer; ML, molecular layer; Py, pyramidal cell layer; SO, stratum oriens; SR, stratum radiatum. Values are shown as the mean ± SD. Asterisks (** and ***) denote statistical significance at $p < 0.01$ and 0.001, respectively, by one-way ANOVA with the Tukey–Kramer *post hoc* test.

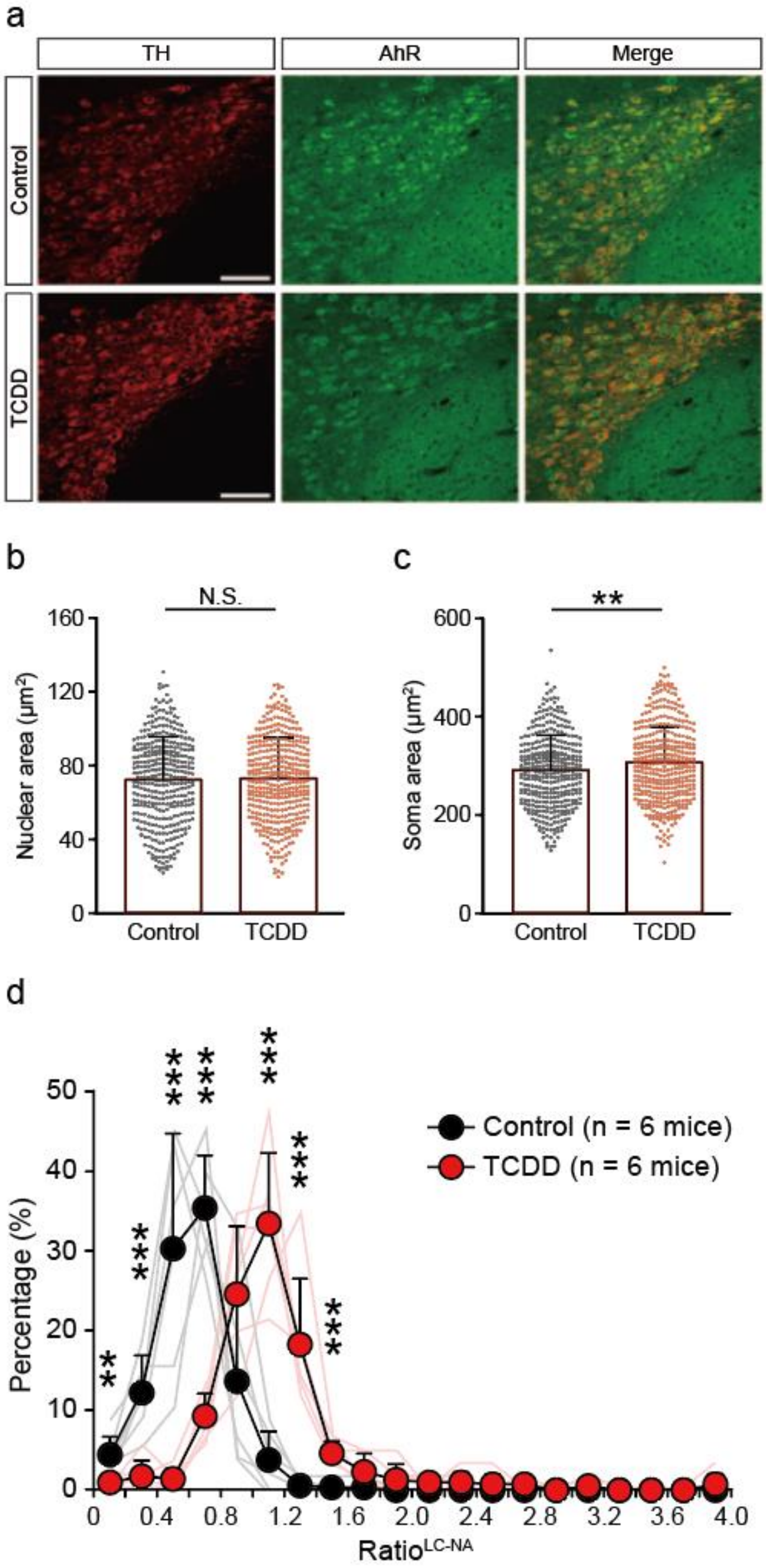
a
TH
AhR
Merge
Control
TCDD
b
N.S.
Nuclear area (μm²)
160
120
80
40
0
Control
TCDD
c
**
Soma area (μm²)
600
400
200
0
Control
TCDD
d
Percentage (%)
50
40
30
20
10
0
0
0.4
0.8
1.2
1.6
2.0
2.4
2.8
3.2
3.6
4.0
Ratio^LC-NA
Control (n = 6 mice)
TCDD (n = 6 mice)

Supplementary Figure 2. AhR expression and ratio$^{LC\text{-}NA}$ percentage values of LC-NA neurons in TCDD-exposed mice. (a) Representative images of the immunostained LC in the control and TCDD groups. Scale bars = 100 μm. (b, c) Nuclear area (b) and soma area (c) of LC-NA neurons in the control and TCDD groups. Although the soma area in the TCDD group was significantly higher than that in the control group (c), the nuclear area (b) and nuclear area percentage (Figure 7b) did not differ, suggesting the absence of severe injury at the cellular level in TCDD-exposed LC-NA neurons. Circles represent individual cell data (382 and 398 cells from 6 mice each in the control and TCDD groups, respectively). (d) Distribution of nuclear AhR amounts, represented as ratio$^{LC\text{-}NA}$ percentage values in each mouse, in the control (black) and TCDD (red) groups. The ratio$^{LC\text{-}NA}$ percentages were shifted toward higher values in the TCDD group (peak value = 1.0–1.2) compared with the control group (peak value = 0.6–0.8). Ratio$^{LC\text{-}NA}$ percentages that range from 0 to 1.6 are arbitrarily divided by every 0.2 were significantly different between the two groups. Circles and lines represent the average percentage at each ratio$^{LC\text{-}NA}$ and individual mouse data, respectively (n = 6 mice/group). Values are shown as the mean ± SD. Asterisks (** and ***) denote statistical significance at $p < 0.01$ and 0.001, respectively, by Student's *t*-test.

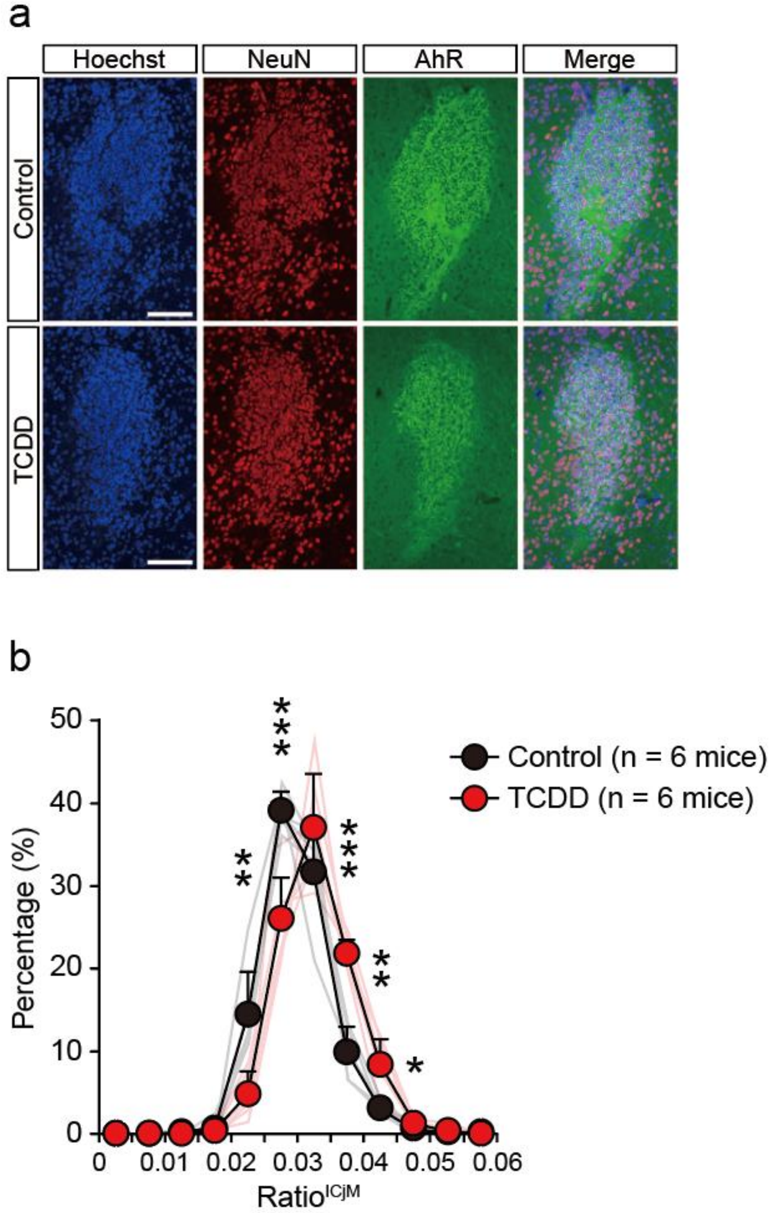

Supplementary Figure 3. AhR expression and ratio^{ICjM} percentage values of ICjM neurons in TCDD-exposed mice. (a) Representative images of the immunostained ICjM in the control and TCDD groups. Scale bar = 100 µm. (b) The distribution of ratio^{ICjM} values in the control (black) and TCDD (red) groups. The ratio^{ICjM} percentages shifted toward higher values in the TCDD group (peak value = 0.030–0.035) compared to the control group (0.025–0.030). Ratio^{ICjM} percentages ranging from 0.02 to 0.05 were arbitrarily divided by every 0.005 differed significantly between the two groups. Circles and lines represent the average percentage at each ratio^{ICjM} and individual mouse data, respectively (n = 6 mice/group). Values are shown as the mean ± SD. Asterisks (*, **, and ***) denote statistical significance at $p < 0.05$, 0.01, and 0.001, respectively, by Student's *t*-test.
